# Exploring How Accountability Affects the Medical Decisions We Make for Other People

**DOI:** 10.3389/fpsyg.2019.00079

**Published:** 2019-02-08

**Authors:** Eleonore Batteux, Eamonn Ferguson, Richard J. Tunney

**Affiliations:** ^1^School of Psychology, University of Nottingham, Nottingham, United Kingdom; ^2^Department of Psychology, Aston University, Birmingham, United Kingdom

**Keywords:** surrogate decision-making, self-other differences, accountability, medical decision-making, DMfO

## Abstract

In the event that a patient has lost their decision-making capacity due to illness or injury, a surrogate is often appointed to do so on their behalf. Research has shown that people take less risk when making treatment decisions for other people than they do for themselves. This has been discussed as surrogates employing greater caution for others given the accountability they are faced with. We tested the prediction that making accountability salient reduces risk-taking for others relative to the self by manipulating the information shown to participants while they made treatment choices. One group was asked to focus on the consequences for the recipient’s family, another on the legal implications of their decisions, and another was not given additional information. Participants reduced their risk-taking for others compared to themselves, irrespective of the condition they were in. Although participants in each condition reported thinking about these factors to different extents, there were no clear differences in risk-taking between groups. However, we did find that, across all participants, thinking about legal consequences reduces risk-taking. We suggest that future research investigates how the effect of thinking about accountability on surrogate choices is mediated by feelings of accountability, in order to further examine the explanations suggested in the literature.

## Introduction

Making decisions on behalf of someone else is not an easy task and often places a high level of responsibility on the decision-maker, particularly in a medical context where the life of a patient is at stake. In the event that a patient is unable to make a decision for themselves due to illness or injury, medical decisions are often made by a surrogate in cases where the patient has not made a legally binding advance directive^1^. In the United States for example, family members are often burdened with making decisions in the intensive care unit, where only about 5% of patients are deemed able to make decisions for themselves (Radwany et al., 2009). If knowledge of the patient’s wishes is available, the surrogate is instructed to make a decision that follows the substituted judgment standard – i.e., make the decision the recipient would have made for themselves, thereby putting aside their own wishes and preferences for the patient. On the other hand, in the absence of information about a patient’s wishes, the best interests standard is followed – i.e., the best possible outcome that provides the most benefit for the patient. Are surrogates able to take decisions that accurately represents the recipient’s wishes and preferences, or are they influenced by other factors?

Qualitative research highlights that surrogates feel conflicted between making a decision that reflects the patient’s wishes and factors such as preserving the patient’s life or the family’s well-being (Schenker et al., 2012; Dionne-Odom et al., 2015; Fetherstonhaugh et al., 2017). In terms of whether surrogates are actually capable of predicting their next-of-kin’s treatment preferences, a systematic review showed that they were accurate only 68% of the time (Shalowitz et al., 2006). Interestingly, it seems that surrogates are biased toward predicting that a patient would want to be treated and are therefore more accurate in cases where the patients are favorable to treatment (Frey et al., 2014). Moreover, surrogates seem to have preferences regarding the procedure that should be followed when making a surrogate decision, which might in turn affect whether the substituted judgment is adequately followed (Frey et al., 2018). Taken together, these findings show that the substituted judgment standard is unlikely to be met in most cases, thereby adding to the debate concerning its suitability (Torke et al., 2008). What about the cases where surrogates do not know the wishes of the patient? In this paper, we theorize that the accountability placed on a surrogate will come into play and explore its influence on surrogate decisions in treatment scenarios involving risk.

For the purpose of this paper, we adopt a rather broad definition of accountability which refers to the answerability a decision-maker has – i.e., the responsibility for justifying their decisions. Accountability can manifest itself in a number of more specific ways. From a legal perspective, accountability would refer to being answerable to a court of law in the event that the decision-maker is accused of making an incriminating decision. This would presumably push the decision-maker toward making a decision that would not incriminate them. The decision-maker can also be held accountable in a more indirect manner – by the recipient themselves or the recipient’s family for the harmful consequences of the decision. It is conceivable that this would push the decision-maker toward an empathic response that aims to minimize potential harm to the recipient and their family. We aim to investigate how consideration of these factors influences surrogate treatment decisions.

When doctors are faced with hypothetical scenarios in which they have to make treatment decisions or recommendations for their patients, research shows that they accept less risk for their patient than they do for themselves (Ubel et al., 2011; Garcia-Retamero and Galesic, 2012a; Janssen et al., 2015). Ubel et al. (2011) argue that these differences arise due to an effect of accountability whereby physicians feel the need to be able to justify their choices to others. Garcia-Retamero and Galesic (2012a) showed that doctors report that they fear the legal consequences of their decisions and thereby reduce the risk they are prepared to take for a patient relative to themselves. They also found that doctors did not make decisions that were in line with their predictions of the patient’s risk preferences. These results highlight the role accountability plays in the way doctors reduce their risk-taking for their patients relative to themselves, which is not surprising given that litigation against medical professionals is on the rise (Garcia-Retamero and Galesic, 2012b).

However, this reduction in risk-taking is not exclusive to doctors. It has also been found when people from the general population make decisions for a hypothetical patient (Zikmund-Fisher et al., 2006; Oliver, 2013), a family member (Zikmund-Fisher et al., 2006; Petrova et al., 2016; Tang et al., 2016; Carroll et al., 2017) or a stranger (Batteux, unpublished). This has been interpreted as surrogates being more cautious when deciding for someone else (Oliver, 2013), as well as stemming from the need to justify one’s decisions (Zikmund-Fisher et al., 2006), in which case maximizing survival chances is easier to defend. It has also been shown that this reduction in risk-taking is apparent even when it goes against the recipient’s preferences (Petrova et al., 2016). Overall, it seems that this effect occurs regardless of the identity of both the decision-maker and the recipient. Even though professional accountability is not relevant in the case of ordinary decision-makers, it is plausible that some other form of accountability is responsible for the reduction in risk-taking, such as the responsibility the decision-maker has toward the recipient and their family to make a well-founded decision. In fact, similar accounts have been put forward when discussing the discrepancies between our own choices and our advice to others – we are more cautious when advising others to avoid being responsible for their loss (Dana and Cain, 2015). Our aim here is to explore the role accountability plays when ordinary decision-makers make surrogate treatment choices.

Past research has often focused on scenarios that speak of the possibility of the patient dying, either without treatment (Zikmund-Fisher et al., 2006; Ubel et al., 2011; Garcia-Retamero and Galesic, 2012b; Oliver, 2013; Tang et al., 2016) or as a consequence of treatment (Ubel et al., 2011; Carroll et al., 2017; Batteux et al., unpublished). Crucially, in all of these cases surrogate decisions were directed toward the option that reduced the patient’s likelihood of dying. Wanting to preserve the patient’s life and give them a chance is clearly apparent in qualitative reports by surrogates (Schenker et al., 2012; Dionne-Odom et al., 2015; Fetherstonhaugh et al., 2017). This often constitutes the reason why surrogates have such a difficult time deciding and might be prevented from acting in accordance with the recipient’s values. We therefore hypothesize that self-other differences are driven by the wish to increase the patient’s likelihood of survival, over and above other potential costs (such as diminished quality of life). For that reason, we expect that accountability pushes surrogates toward making a decision that preserves the patient’s life.

The predictions made regarding accountability are supported by current theories of surrogate decision-making. Tunney and Ziegler’s (2015) model proposes that surrogate decisions are the result of perspective taking that varies according to the features of the decision. In the context of medical decisions, a next-of-kin might engage in a simulated perspective, thereby making the decision the patient would have wanted (i.e., follow the substituted judgment standard), whereas a doctor might adopt a more benevolent perspective and make a decision that is in the patient’s best interest (i.e., follow the best-interests standard). On the other hand, the accountability held against the decision-maker is likely to also make them engage in an egocentric perspective where they consider what is best for themselves, which might prevent them from making a simulated or a benevolent decision. Medical professionals might do this if they fear the professional or legal consequences of making the wrong decision, in which case it would be easier for them to justify a decision that is aimed at saving lives. Ordinary decision-makers might adopt an egocentric perspective if they fear going against the family’s wishes, even if that might mean overriding what the patient would want. This coincides with qualitative reports which show that surrogate decision-makers struggle to reconcile the family’s wishes with what the patient would want (Schenker et al., 2012; Dionne-Odom et al., 2015; Fetherstonhaugh et al., 2017).

Additionally, Social Values Theory (Stone and Allgaier, 2008) proposes that surrogate decisions are made according to the social value placed on taking or avoiding a risk. It has indeed been found that surrogate health and safety decisions are made in line with what people perceive to be a socially acceptable level of risk-taking (Stone et al., 2013). If taking risks in a medical setting is frowned upon, it makes sense that surrogates would want to minimize risk-taking to avoid being blamed for the negative consequences of their decision, and it is even more likely that surrogates take this into account when held accountable for their decision.

In this study, we examined the hypothesis that once we make the accountability for the negative consequences of taking a risk (i.e., death) salient, decision-makers reduce their risk-taking for others relative to themselves. Although the literature discusses findings in this way, this interpretation has not been formally tested with ordinary decision-makers. Specifically, we want to understand whether the self-other differences that have been reported have arisen due to surrogates thinking about their own accountability. We theorized that there might be two different sources of accountability that have an impact on surrogate risk-taking: the recipient’s family and the potential legal implications of making a decision that threatens a patient’s life. In doing so, we can assess whether the fear of legal repercussions is also relevant to ordinary decision-makers. Given that we expect that the main source of accountability experienced by surrogates relates to the recipient’s survival, we focused our accountability manipulation on the eventuality that the decision leads to the death of the recipient.

We tested the impact that accountability salience can have on risk-taking by manipulating the information we presented to participants when making their surrogate decisions. We predict that making these accountability factors more salient to participants will further decrease their risk-taking. To assess self-other differences in choices, we used the QALY (quality-adjusted life years) standard gamble (SG) method commonly used to measure the utility of health states (Whitehead and Ali, 2010). It measures utility under risk for a particular medical condition by presenting a choice between a safe option^2^ (staying in that condition) and a risky option (taking a risky treatment which could lead to the death of the patient). We used both relatively minor and severe illnesses to investigate whether accountability salience had the same effect on both.

## Materials and Methods

### Design

A 2 (Recipient) × 2 (Magnitude) × 3 (Accountability) mixed design was used. “Recipient” was a within-subjects factor where participants made decisions for themselves (self) and for another participant (other). “Magnitude” was a within-subjects factor relating to the severity of the health state. “Accountability” was a between-subjects factor which refers to how accountability was made salient to participants (control, family, legal).

### Participants

Participants (*n* = 86) were recruited from the University of Nottingham. Two participants were excluded because they did not understand the task (one misinterpreted the choices and the other repeatedly pressed the wrong keys). The sample size was determined using G^∗^Power 3.1 (Faul et al., 2007). We expected an interaction between recipient and accountability. Given that this is the first study to test the effects of accountability on self-other differences in medical decisions, we could not compute an effect size based on previous research and therefore theorized that we would find a small to medium effect size. A sample size of 84 enables the detection of a small to medium effect size (*d* = 0.35) with adequate power (>0.80) and an acceptable alpha level (<0.05). The age group ranged from 18 to 34 (*M* = 20.65, *SD* = 3.31). There were 21 males and 65 females. Ethical approval was obtained from the University of Nottingham ethics committee.

### Choice Task

Participants completed the experiment on a computer using PsychoPy (Peirce, 2007). They were presented with six illness scenarios (three large magnitude scenarios and three small magnitude scenarios, see Appendix 1). The order in which they were presented these scenarios was randomized. Each participant completed each scenario twice: once for themselves and once deciding for another unknown participant. The order in which they were presented with each recipient condition was counterbalanced across participants. They were told that the other participant was a student of a similar age and situation to them. Participants were given the choice between a safe option: remaining in a condition (paraplegia, Broca’s aphasia, vegetative state, angina, headache, nausea) and a risky option: a treatment with a probability *p* of a complete recovery and a probability 1 – *p* of death. The probability *p* in the risky option was presented in descending order (100, 95, 90, 80, 70, 60, 50, 40, 30, 20, 10, 5, and 0%) until respondents switched from choosing the risky option to the safe option. Instructions and example trials can be found in Appendix 1.

### Accountability Salience

In the family accountability condition, participants were asked to think about the recipient’s family: “In the event of that person’s death, their family will be devastated. Consider how the family would feel and think carefully about the consequences your choice would have for them before you make it.” In the legal accountability condition, participants were told what the legal consequences of their decision might be: “In the event of that person’s death, you will be held legally responsible for it. If you are able to justify your choice, you will not be prosecuted. Think carefully about your choice before you make it.” In the control condition, participants were not provided with additional information or instructions. Accountability was only made salient to participants in the other condition, not in the self-condition.

### Manipulation Checks

In order to check whether making accountability salient had an effect, all participants were presented with two questions relating to their surrogate choices at the end of the task. One asked whether they thought of the consequences the recipient’s death might have for their family, and the other whether they thought of the legal consequences that the recipient’s death might have for them. Participants responded on a 5-point Likert scale (1 being “not at all” and 5 being “a great deal”).

## Results

In line with previous literature, we computed participants’ utilities of the medical scenario for each recipient (Whitehead and Ali, 2010). We did so by taking their indifference point between taking the risky option and the safe option. The indifference point is the average of the two probabilities each side of the crossover point from the risky to the safe option. Utilities varied between 0 and 1 where 0 indicates that they always chose the risky option and 1 indicates they always chose the sure option. We then averaged utilities for large magnitude scenarios and small magnitude scenarios to have an overall utility for each condition. The utilities for each recipient and outcome magnitude can be found in Table 1 by accountability salience condition. We checked whether these utilities were normally distributed and met the criteria to be entered in an analysis of variance (ANOVA). Their distribution can be found in Appendix 1.

**Table 1:** Mean utilities with standard deviations across participants for each recipient and outcome magnitude by accountability salience condition.

	Self		Other	
	Large	Small	Large	Small
Control	0.55 (0.19)	0.76 (0.17)	0.67 (0.19)	0.78 (0.17)
Family	0.46 (0.20)	0.73 (0.17)	0.63 (0.18)	0.78 (0.17)
Legal	0.47 (0.21)	0.70 (0.19)	0.62 (0.19)	0.76 (0.16)


**FIGURE 1:**
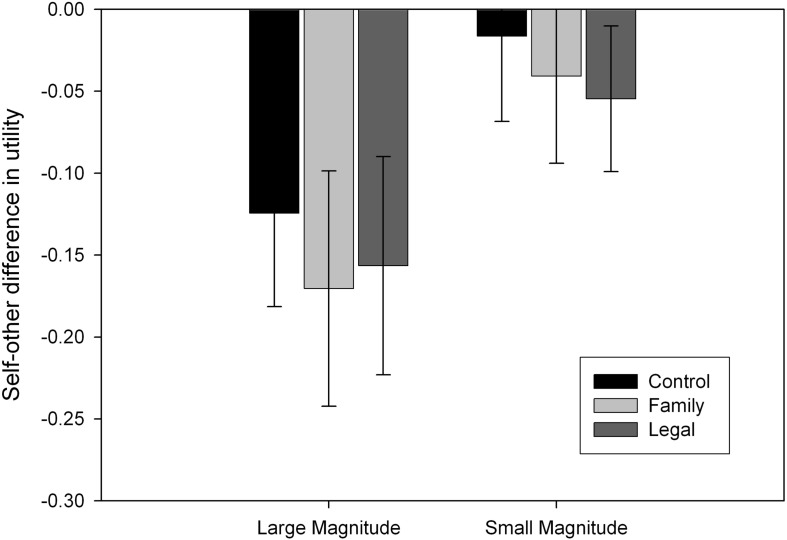
Self-other differences for each outcome magnitude per condition, with error bars indicating 95% confidence intervals. Negative values indicate that participants took more risk for themselves than for someone else.

We entered these utilities in a 2 (Recipient) × 2 (Magnitude) × 3 (Accountability) mixed model ANOVA where recipient (self, other) and outcome magnitude (large, small) were within-subject factors and accountability (control, family, legal) was a between-subject factor. There was a main effect of recipient: participants were more risk-averse for someone else than for themselves (*F*_1,81_= 41.90, *MSE* = 0.02, *p* < 0.001, $\eta_{p}^{2}$ = 0.341). There was a main effect of magnitude: participants were more risk-averse for small than for large magnitudes (*F*_1,81_= 149.95, *MSE* = 0.02, *p* < 0.001, $\eta_{p}^{2}$ = 0.649). There was also an interaction between recipient and magnitude (*F*_1,81_= 52.731, *MSE* = 0.01, *p* < 0.001, $\eta_{p}^{2}$ = 0.405): self-other differences were greater with large magnitudes (mean difference = –0.150, *p* < 0.001) than with small magnitudes (mean difference = –0.037, *p* = 0.010) according to a simple effects analysis. However, there were no interactions with accountability salience. Figure 1 shows the self-other difference for each outcome magnitude per condition.

To check for any order effects relating to the recipient condition, we ran the analysis again with the order factor (self-other, other-self). The above results remained the same, with the addition of an interaction between recipient and order (*F*_1,78_= 8.385, *MSE* = 0.017, *p* = 0.005, $\eta_{p}^{2}$ = 0.097). According to a simple effects analysis, self-other differences were larger when the self-condition was completed first (mean difference =–0.133, *p* < 0.001) rather than the other condition completed first (mean difference = –0.052, *p* = 0.011). This reinforces the need for conditions to be counterbalanced to control for order effects.

To further investigate the null effect of accountability salience, we conducted a Bayesian mixed model ANOVA. We did not find evidence for an effect of accountability salience. Full details of these results can be found in Appendix 1.

**FIGURE 2:**
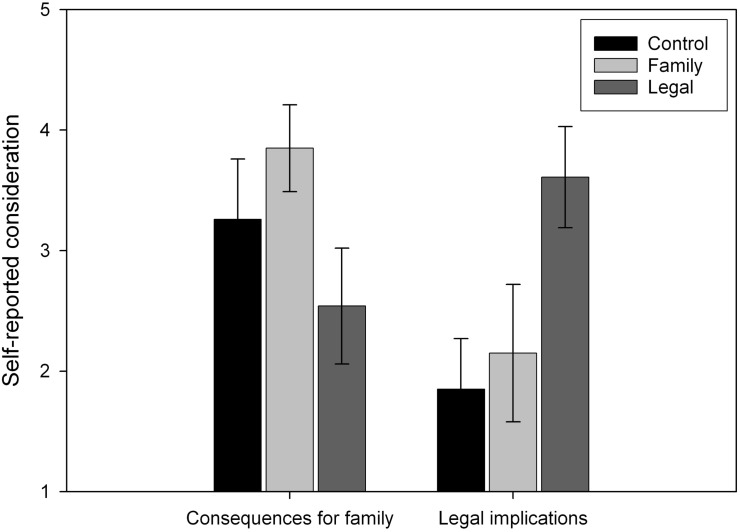
Participants’ reports of how much they thought about the recipient’s family and legal consequences while making a surrogate choice. Higher values indicate that participants thought about a particular factor to a greater extent. Error bars represent 95% confidence intervals.

The manipulation checks showed that participants in each condition thought about different factors when making the surrogate decision (see Figure 2). Participants’ responses were entered in a 2 (Factor) × 3 (Accountability) mixed model ANOVA. There was a main effect of factor: participants reported thinking more about the recipient’s family than about legal consequences (*F*_1,79_ = 13.99, *MSE* = 1.35, *p* < 0.001, $\eta_{p}^{2}$ = 0.150). There was also an interaction between factor and accountability condition (*F*_1,79_ = 23.706, *MSE* = 1.35, *p* < 0.001, $\eta_{p}^{2}$ = 0.375). According to simple effects analyses, participants in the control condition thought more about the recipient’s family than legal consequences (mean difference = 1.407, *p* < 0.001), as did participants in the family condition (mean difference = 1.704, *p* < 0.001). Participants in the legal condition, however, thought more about legal consequences than the recipient’s family (mean difference = 1.071, *p* = 0.004).

We investigated whether there was a relationship between self-other differences overall and self-reports of the extent to which participants thought about a particular factor using Pearson’s correlations. There was no relationship between the family factor and self-other differences (*r* = 0.014, *p* = 0.903), which would make sense if people were also thinking about their families when deciding for themselves. On the other hand, there was a positive relationship between the legal factor and self-other differences (*r* = 0.248, *p* = 0.025), which again would make sense given that legal responsibilities would not apply when making decisions for oneself. It seems that thinking about legal consequences decreases risk-taking for other relative to the self.

## Discussion

Surrogates were less willing to accept a treatment that carried a risk of dying for another person as they did for themselves. This is consistent with the literature which has found that surrogates are more likely to make choices that avoid a risk of death for others than for themselves (Zikmund-Fisher et al., 2006; Ubel et al., 2011; Garcia-Retamero and Galesic, 2012a; Oliver, 2013; Petrova et al., 2016). These findings support accounts of self-other differences based on caution due to the responsibility placed on the decision-maker, but also given the uncertainty contained in deciding in the absence of knowledge of the recipient’s preferences in our study. Notably, we found that self-other differences held across both magnitudes, meaning that decision-makers reduced their risk-taking for others both when considering minor and severe illness scenarios.

Our findings suggest that the accountability manipulation did not significantly affect surrogate’s propensity to accept the risky treatment, even though the manipulation checks show that it did have an effect on participants’ thought process. It seems that in the family condition, participants’ attention was guided toward thinking about a factor that those in the control condition considered anyway, but that participants in the legal condition were steered away from it and toward a different factor. Our assumption here was that by emphasizing to participants the factors that we expect drive self-other differences, this would further decrease surrogate risk-taking. Our prediction was not supported in our main analysis, although we do observe a trend which indicates that it might have been detected as a small effect in a higher powered study. We also found tentative evidence which does not exclude the possibility that accountability influences self-other differences. The more participants reported thinking about legal consequences, the more likely they were to reduce their risk-taking for the recipient relative to themselves. However, participants in both the control and family condition do not seem to take legal consequences into much consideration, meaning that self-other differences in those groups cannot be explained by that. On the other hand, they both report taking the recipient’s family into consideration. The fact that participants in the control group spontaneously thought about the family factor suggests that it could be responsible for the reduction in risk-taking observed in the literature that is not specific to doctors, rather than any legal consequences.

Interestingly, we found a discrepancy between the thoughts our participants and doctors spontaneously report, even though in both cases thinking about legal repercussions seems to reduce risk-taking (Garcia-Retamero and Galesic, 2012b). Contrary to the case of doctors, our participants did not seem to take the legal consequences into much consideration, which makes sense given the strong professional responsibilities that affect doctors. However, we still find similar self-other differences, which suggests that multiple factors lead to a reduction in risk-taking. This could be indicative of a strong norm for taking less risk for others in a medical context and lends support to the idea that these decisions are made according to the social value placed on risk-taking (Stone and Allgaier, 2008). Moreover, if accountability drives these self-other differences, it is conceivable that it is in fact the social norm which is steering the effect of accountability. Given that we define accountability as the need to justify one’s decisions to others, it would make sense to rely on a social norm to do so. In the case of doctors for example, they might rely on the social norm that they are expected to save lives. The influence of social norms on the effect of accountability on self-other differences is an interesting empirical question which remains open to investigation.

It is important to note that we conceptualized risk-taking in this study as the option that carries the risk of the recipient dying. However, refusing treatment is also an option that carries a risk – i.e., remaining ill might lead to harm further down the line. Although the scenarios specifically laid out the symptoms and living conditions associated with the illness, it is conceivable that participants also considered the safer option to be risky. Relatedly, this could mean that they would also feel accountable for not taking the treatment and leaving the recipient with the illness. The fact that risk presents itself in both the safe and risky options, favoring the safe option might simply mean that participants are avoiding the risk of dying, rather than being risk-averse. Considering that participants chose the status quo (i.e., the safe option) for someone else more often than for themselves, this shows further support for our hypothesis that surrogates favor the option that maximizes the recipient’s survival regardless of whether this entails taking or avoiding treatment. This is consistent with previous research that shows that doctors are more likely to take a treatment with a higher risk of death but a lower risk of complications for themselves than they are for a patient (Ubel et al., 2011). In light of this, self-other differences in treatment scenarios could be reinterpreted as surrogates being more likely to favor life preservation for others, at the expense of their quality of life, than they are for themselves. In that case, decisions might not be consistently less risk-taking for others, but instead seek the option that prevents the risk of the recipient dying.

Relatedly, by making the treatment option the risky option and the status quo the safe option, we introduced a confound. Perhaps participants were more likely to choose the status quo for others rather than more likely to choose the safer option, out of fear of being accountable for interfering with the natural course of events for example. However, studies that made the treatment option the safe option and the status quo the risky option also found that participants favored the safe option for others (Zikmund-Fisher et al., 2006; Ubel et al., 2011; Petrova et al., 2016; Tang et al., 2016). This sheds light on the confound in the present study and suggests that the explanation we propose holds for cases where the risky option is either taking or refusing treatment. Furthermore, it is possible that the design of our experiment encouraged risk-seeking behavior by making the treatment option the default option (which starts out as being a safe option with no risk of death)^3^. Indeed, the default literature would predict that people are more likely to stick to the default option (Johnson and Goldstein, 2003). This should not have impacted self-other differences unless decision-makers are less likely to stick to the default option for others, which is an interesting question for the wider decision-making literature. Finally, future studies should keep in mind that it might be more ecologically valid to have the status quo as the default option as opposed to the treatment.

### Avenues for Future Research

Crucially, what we did not measure here was the participants’ feelings of accountability, both in terms of their own guilt and responsibility and their fear of the potential repercussions for them. It is conceivable that it is the emotional response to thinking about these factors that drives the reduction in surrogate risk-taking, rather than the mere fact that participants consider them. Perhaps most participants would think about these factors, but not all would be swayed by them when making their decision. Moreover, making accountability salient did not alter the specific scenarios but rather pushed participants to think about their accountability as the decision-maker. Perhaps a more effective way of testing the effect of accountability would be to compare scenarios that include elements that specifically increase the accountability held against the decision-maker (e.g., the decision-maker is convicted if the recipient dies) or decrease it (e.g., the decision-maker is guaranteed anonymity). Finding more sophisticated ways of assessing accountability is an important step for future research to understand its role.

Investigating an unknown other as the recipient allows investigation of cases where the surrogate has to decide in the absence of information about the recipient’s wishes. Nevertheless, it would be worth investigating whether the thought process changes when the surrogate is aware of the patient’s wishes. Perhaps feelings of accountability diminish when the patient’s wishes are clear and respected by the surrogate. Given that the surrogate would not know the wishes of the recipient’s family either, it remains open to question whether they conceptualized the wishes of the family to be different to what we emphasized to them in the scenarios. It would be interesting to investigate whether surrogates would hold different assumptions concerning the wishes of a patient’s family.

## Conclusion

We found that participants were more likely to refuse a treatment that carries a risk of death for someone else than for themselves, therefore implying that they would rather leave them ill than risk their death. It is conceivable that previous findings can be reinterpreted as surrogates favoring saving lives for others more so than for themselves, rather than necessarily taking more risks for themselves than for others. We explored the idea that this was due to participants being driven by the thought of being held accountable in the event of the recipient’s death. Our findings show tentative evidence that thinking about accountability steers surrogates away from risking the recipient’s life, but further research is necessary. However, we did find that participants considered the repercussions for the recipient’s family, and at times legal repercussions when making a surrogate decision. This suggests that participants are considering multiple factors, although it is still unclear how they affect the decisions they make. These findings can speak to the reality of surrogate decision-making, which often involves a struggle to reconcile the patient’s wishes with a multitude of other perspectives and responsibilities (Schenker et al., 2012; Dionne-Odom et al., 2015; Fetherstonhaugh et al., 2017). This supports the idea that a surrogate decision involves a lot more than fulfilling the substituted judgment standard. Asking surrogates to put themselves and the recipient’s family aside appears to be an unrealistic expectation.

## Ethics Statement

This study was carried out in accordance with the recommendations of The British Psychological Society Code of Conduct with written informed consent from all subjects. All subjects gave written informed consent in accordance with the Declaration of Helsinki. The protocol was approved by the University of Nottingham, School of Psychology Ethics Committee.

## Data Availability

We have made the data publicly available on https://osf.io/nckxw/.

## Author Contributions

EB conducted the data collection and analysis, and wrote the first draft of the paper. RT and EF are supervised the study.

## Conflict of Interest Statement

The authors declare that the research was conducted in the absence of any commercial or financial relationships that could be construed as a potential conflict of interest.

## Footnotes

1 The specific instances and circumstances in which surrogate decisions are made vary depending on a country’s legislation, thereby making more or less common from one country to the next.

2 Although we label this option the “safe” option to distinguish it from the “risky” option in our study, the “safe” option can be thought of as carrying risks as well, which we will address in our discussion.

3 We included this trial to check that participants were paying attention and responding honestly, under the assumption that they would take a treatment which lead to a full recovery. Note that participants always selected that option so we did not exclude any on that basis.
