# Supplementary material for: Exploring How Accountability Affects the Medical Decisions We Make for Other People
Source: Front Psychol. 2019 Feb 8;10:79. doi: 10.3389/fpsyg.2019.00079 (PMC6375882; doi:10.3389/fpsyg.2019.00079)
Supplement: Supplementary file 1 [file Table_1.doc]

Appendix 1: Medical scenarios[[1]](#footnote-2)

*Large magnitude*

**Paraplegia**: Imagine you suddenly develop paraplegia. You suffer from a complete loss of sensation and movement from the waist down, meaning that your legs are paralysed. You are wheelchair-bound and you lose control of your bladder and your bowel. You require some assistance with self-care. Without treatment, there is no chance of recovery.

**Broca’s Aphasia**: Imagine that you suddenly develop Broca’s aphasia (a type of stroke). You will have a very difficult time speaking and writing. Nobody can understand you except for maybe the one or two people closest to you. This is very frustrating because you can understand other people but they don’t understand you. You can speak a little but you have to work hard at it and most of the words you say will not make sense. Without treatment, there is no chance of recovery.

**Vegetative State**: Imagine you are the victim of an accident which leaves you in a vegetative state. You are bed-bound and need to be fed through a tube. You have almost lost all consciousness. You are unable to see, speak, and can barely think. You can hear and understand some of the things that are going on around you. You can slightly move your fingers meaning that you can occasionally communicate. There is no pain with this condition and without treatment, there is no chance of recovery.

*Small magnitude*

**Angina**: Imagine that you suddenly develop an angina. You have pain or discomfort in mainly in your chest but also in your upper body. You suffer from fatigue and occasional dizziness. You find breathing more difficult than usual. You can take medication to alleviate the symptoms but these are persistent and without treatment, there is no chance of recovery.

**Headache**: Imagine that you suddenly develop a persistent headache. You suffer from an aching head pain and a sensation of tightness or pressure across your forehead. You struggle to concentrate and the pain can render you unable to take part in your daily activities. Medication does not entirely relieve the pain and without treatment, there is no chance of recovery.

**Nausea**: Imagine that you develop an illness with symptoms of nausea. You often feel discomfort in your stomach and you are occasionally sick. This makes it more difficult for you to eat and get on with your day. You may be unable to work if the discomfort is too high. You can take medication to reduce the symptoms and without treatment, there is no chance of recovery.

Appendix 2: Instructions and example trial


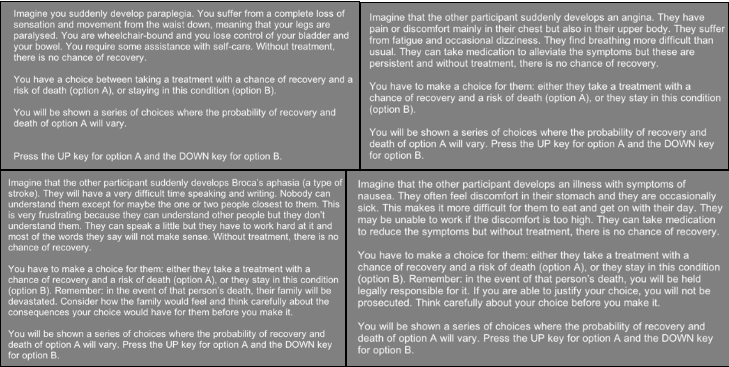


Figure 3: Instructions shown to participants for each accountability manipulation (self, other control, other family, other legal).


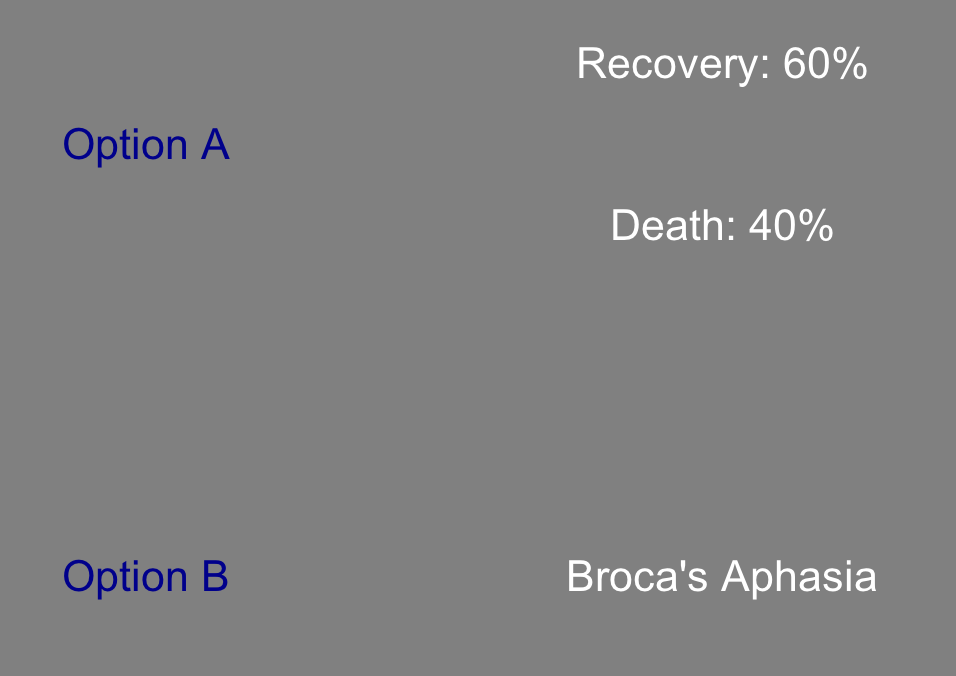


Figure 4: Example trial for the Broca’s aphasia scenario.

Appendix 3: Distributions of each recipient and magnitude across accountability manipulations


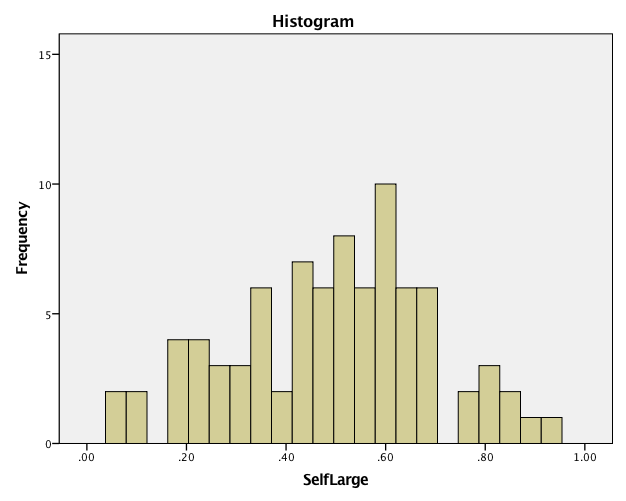


Figure 5: Distribution of utilities for self for large magnitude.


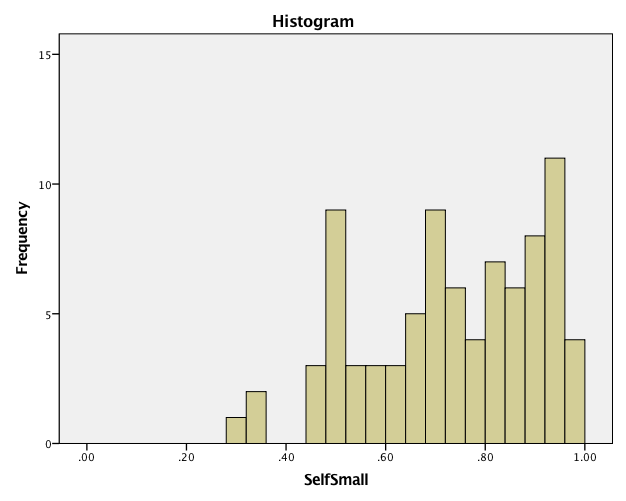


Figure 6: Distribution of utilities for self for small magnitude.


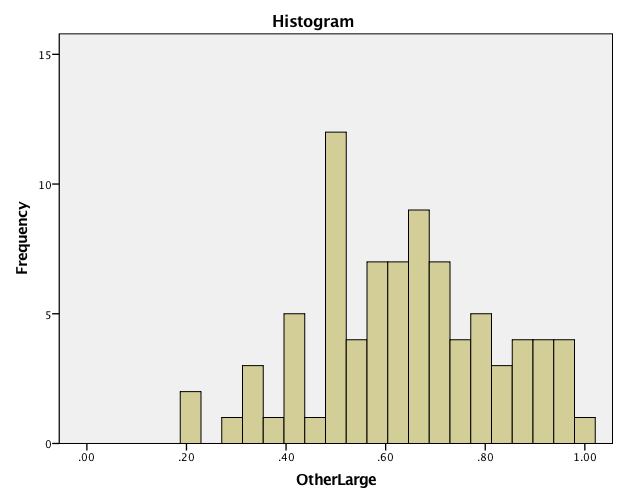


Figure 7: Distribution of utilities for other for large magnitude.


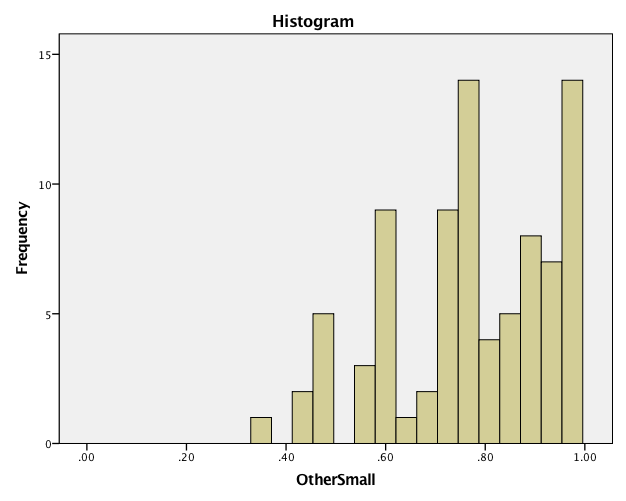


Figure 8: Distribution of utilities for self for small magnitude.

Appendix 4: Bayesian analysis of effects

| Effects | BF_Inclusion_ |
| --- | --- |
| Recipient | 1.358*10^11^ |
| Magnitude | 3.217*10^15^ |
| Accountability | 0.135 |
| Recipient*Magnitude | 2194.166 |
| Recipient* Accountability | 0.074 |
| Magnitude* Accountability | 0.087 |
| Recipient*Magnitude* Accountability | 0.009 |

1. The scenarios presented are worded as in the *self* condition. In the *other* condition, it was made clear that the other was another participant and all pronouns were changed accordingly. [↑](#footnote-ref-2)
